# Muti-factor analysis of sport activity level after high tibial osteotomy

**DOI:** 10.1186/s13018-023-04305-3

**Published:** 2023-10-31

**Authors:** Teng Huang, Kai Kang, Qi Qiao, Tong Li, Tao Liu, Chenni Ji, Shijun Gao

**Affiliations:** 1 Department of Orthopedic Surgery, Hebei Medical University Third Hospital, Shijiazhuang, Hebei China; 2 Department of Orthopedic Surgery, Hebei General Hospital, Shijiazhuang, Hebei China

**Keywords:** Knee osteoarthritis, High tibial osteotomy, Multi-factor analysis, Sport activity level, General population

## Abstract

**Background:**

Although many studies have shown that high tibial osteotomy is appropriate for active patients, there are limited multifactorial studies on patients’ sport activity level after HTO in general population.

**Methods:**

158 patients who underwent HTO for knee osteoarthritis between January 2016 and December 2019 are included, with a 36-month follow-up. Information was collected from X-rays and questionnaire. The independent variables were age, sex, breadwinner (provide more than 50% income), sport activity level when the knee was pain-free before and after surgery, concomitant meniscal treatment history, Lysholm knee score, desire level for returning to sports. The 158 cases are divided into three groups according to their sports participation before and after operation, Chi-square tests and ANOVA analysis were adopted to identify the effect of these variables on sport activity level after HTO, and factors with statistical differences and clinical relevancies, or provided by previous research were assessed with the ordinal logistic regression analysis.

**Results:**

According to sport activity level analysis, 28(17.7%) patients were categorized into the sport level-reduced group, 97(61.4%) patients into the sport level-unchanged group, and 33(20.9%) patients into the sport level-improved group. Upon ordinal logistic regression analysis, postoperative MA%, age, BMI, and preoperative Lysholm knee score were statistically significant.

**Conclusions:**

Higher postoperative MA%, younger age, lower BMI, and lower Lysholm score are associate with improvement on activity level after HTO. This finding provides valuable references in operation option and rehabilitation planning.

## Background

There are 14 million patients in the United States with osteoarthritis(OA), and nearly half of them are between 45 and 65 [1]. OA poses significant challenges for middle-aged and elderly individuals, considerably deteriorates their athletic ability and life quality [2, 3]. High tibial osteotomy (HTO) aims to adjust the weight-bearing position of the knee joint and relieve patient’s pain symptoms on the knee by correcting the lower limb force line [4–6]. For its quick recovery and minimized inner joint structure change, HTO is seen as an appropriate option for younger patients and high-level sports participants who are suffering from knee osteoarthritis (KOA) [7]. It once again received attention. However, reviews have indicated that parts of the patients are unable to return to physical activity as preoperative after HTO [8, 9]. Reveal the reasons that prevent patients returning to physical activity can improve the clinical outcomes after HTO [10]. Additionally, postoperative sport activity level, which can directly reflect improvements in patients’ knee condition, is gaining attention in recent years [7–9]. Upon literature review, though we found several studies on factors impacting postoperative sport activity level [11, 12], most of them are limited due to their focus on single index and a narrow population, failing to comprehensively explore the postoperative situation of general population [13]. In this project, we explore multiple factors affecting after-surgery sports activity level, adding before-surgery sport activity level and the patient's desire level for returning to sport, which the author believes may be influential. The study aims to enrich both clinical physicians and patients' understanding of sport activity recovery levels following HTO, and to provide a reference for future rehabilitation exercises.

## Study design

### Patients

This study was approved by the ethics committee at our hospital and patient informed consent was also obtained. This was a single-center, retrospective study of continuous patients with KOA who underwent HTO between January 2016 and December 2019 in Hebei Medical University Third Hospital. All patients with KOA were screened, patients with major medical diseases affecting sport activity recovery before operation, patients underwent bilateral HTO at the same time, and patients enduring diseases in rehabilitation affecting sport activity level were excluded.

### Surgical procedure and postoperative management

The HTO procedure was performed by two experienced surgical specialists in this study, employing the technique of biplanar opening-wedge technique [14]. Preoperative measurements and plans were carried out using X-rays of the full length of both lower limbs and the anteroposterior position of the knee joint. The intraoperative plan aimed to locate mechanical axis on the Fujisawa position [6]. 3 cm below the medial knee joint line was selected as the starting point for the oblique ascending osteotomy. If the intraoperative osteotomy gap exceeded 1.4 cm, autologous iliac bone was usually harvested from the patient for grafting, and fixation was achieved with a TomoFix plate (DePuy Synthes, Zuchwil, Switzerland) [15]. To maintain the stability of the osteotomy, the position of the plate was inclined to as posterior as possible [16]. Intraoperative fluoroscopy monitored the changes in the lower limb load line position. A drain was routinely placed and removed 1–2 days post-op, X-rays were reviewed on the first day after surgery. Partial weight-bearing ambulation was initiated under the protection of a walker. Full weight-bearing was achieved at six weeks post-op.

### Radiological measurement

Descriptive data including name, age, sex and body mass index (BMI) were documented. Measurements of the affected limb's hip–knee–ankle angle (HKA), percentage of mechanical axis(%MA), percentage of mechanical axis (MPTA), joint line convergence angle(JLCA),posterior proximal tibial angle(PPTA), Kallgren–Lawrence osteoarthritis grade(K–L grade) and patellar height(Blackburn–Peel index, BPI) were obtained from pre and post-operative long-leg weightbearing anteroposterior view by using RadiAnt DICOM Viewer (21.2 Medixant,Poznan,Poland).

### Questionnaire survey

After 36 months of follow-up, patients were invited to fill out a questionnaire to collect their perioperative information by two orthopedic surgeons who did not participate in the surgery. The questionnaire included the sport activity level when the knee was pain-free before surgery and postoperative 36-month (Low-levels of sports include activities such as walking, gymnastics training, and golf; medium-levels of sports include activities such as mountain walking, hiking, and climbing; high-levels of sports include activities like tennis, badminton, and running), as well as the Lysholm knee score preoperative and postoperative 36-month, whether the patient was the breadwinner of the family. To understand the relationship between the preoperative desire level for returning to sport and the postoperative sport activity level, the questionnaire divided preoperative desire level for returning to sport into four levels: low, medium, high, and extremely high, which were scored by the patients in person to enhance the accuracy of subjective scoring.

### Statistical analysis

Based on the change in sport activity level before and after surgery, patients were classified into groups of sport level-reduced group, sport level-unchanged group, and sport level-improved group. These three groups were statistically described.

Statistical analyses were performed with SPSS Version 26 (IBM), descriptive statistics was used to analyze patient date, we use mean ± standard deviation to describe continuous variables and frequencies with percentage (%) to express categorical variables. Baseline differences between three groups were identified with the ANOVA or the chi-square test.

Upon the conformation of statistically significant differences in this analysis, we added some clinically or scientifically relevant indicators to further explore these variables’ impact on postoperative sport activity level.

When identifying factor’s influence on outcomes, we use ordinal logistic regression and the validity of ordinal logistic regression model with the test of parallel lines. In all tests, P < 0.05 was considered statistically significant.

## Results

**Fig. 1:**
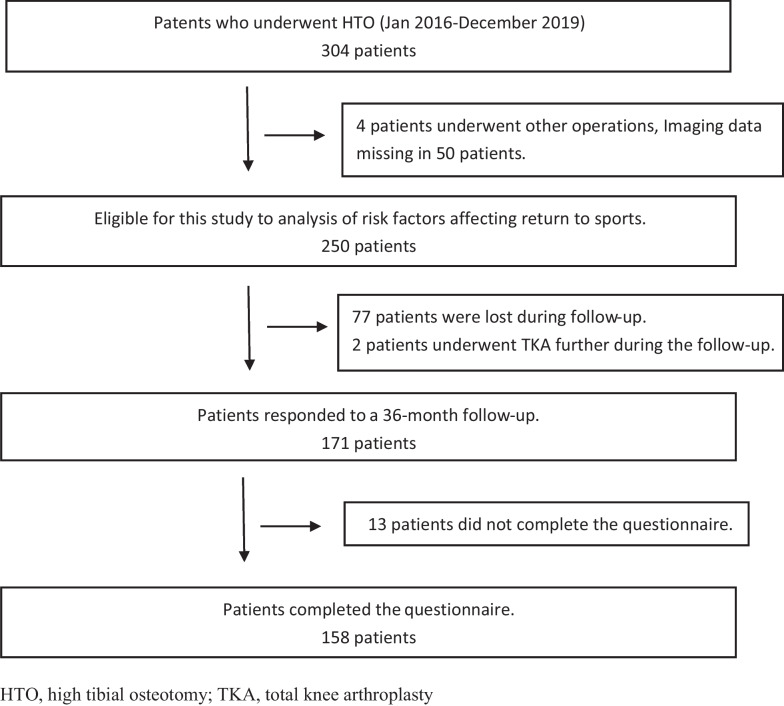
Flowchart of patient inclusion and exclusion

304 patients (62 males and 242 females) with KOA who underwent HTO between January 2016 and December 2019 in Hebei Medical University Third Hospital was admitted. Among them, a total of 250 cases (4 patients underwent other operations, 50 patients’ imaging data were missed) were eligible for this study, 171 cases (77 patients were lost during follow-up, 2 patients underwent TKA during the follow-up) responded to a 36-month follow-up. Apart from 13 patients gave up on the questionnaire, 158 cases completed the questionnaire ultimately (Fig. 1).

The 158 cases are divided into three groups according to their sports participation before and after operation, whose characteristics are presented in Table 1. Of the 158 patients with HTO, 28(17.7%) patients were categorized into the sport level-reduced group, 97(61.4%) patients in the sport level-unchanged group, and 33(20.9%) patients in the sport level-improved group.

**Table 1:** Baseline of clinical variables among the sport level-reduced, unchanged and improved groups

Characteristics	Total (*n* = 158)	Sport level-reduced group (*n* = 28, 17.7%)	Sport level-unchanged group (*n* = 97, 61.4%)	Sport level-improved group (*n* = 33, 20.9%)	*P* value
Age, yr	55.27 ± 6.1	56.18 ± 6.7	55.51 ± 6.4	53.82 ± 4.4	0.269
Sex, *n* (%)					0.436
Male	33(20.9%)	8(28.6%)	20 (20.6%)	5(15.2%)	
Female	125(79.1%)	20(71.4%)	77(79.4%)	28(84.8%)	
BMI, kg/m^2^	27.60 ± 3.9	28.20 ± 6.5	27.51 ± 3.1	27.31 ± 3.34	0.641
Side, *n* (%)					0.858
Left	84(53.2%)	16(57.1%)	50(51.5%)	18(54.5%)	
Right	74(45.8%)	12(42.9%)	47(48.5%)	15(45.5%)	
SAL pre, *n* (%)					< 0.001
Low	102(64.6%)	6(21.4%)	64(66.0%)	32(97.0%)	
Middle	34(21.5%)	12(42.9%)	21(21.6%)	1(3.0%)	
High	22(13.9%)	10(35.7%)	12(12.4%)	0(0%)	
Lysholm pre	58.30 ± 17.5	60.11 ± 18.2	61.65 ± 16.8	46.94 ± 14.0	< 0.001
CMT, *n* (%)					0.275
YES	93(58.9%)	20(71.4%)	53(54.6%)	20(60.6%)	
NO	65(41.1%)	8(28.6%)	44(45.4%)	13(39.4%)	
Preoperative desire level for RTS, *n* (%)					0.794
Low	15(9.5%)	1(3.6%)	14(14.4%)	0(9.5%)	
Medium	14(8.9%)	1(3.6%)	8(8.2%)	5(15.2%)	
High	52(32.9%)	10(35.7%)	35(36.1%)	7(21.2%)	
Extremely high	77(48.7%)	16(57.1%)	40(41.2%)	21(63.6%)	
Breadwinner, *n* (%)					0.916
Yes	47(29.7%)	8(28.6%)	30(30.9%)	9(29.7%)	
No	111(70.3%)	20(71.4%)	67(69.1%)	24(72.7%)	

Continuous variables were described as mean ± standard and categorical variables as frequencies with percentage (%). *BMI* Body mass index, *SAL* Sport activity level, *Lysholm* Lysholm knee score, *CMT* Concomitant meniscal treatment, *RTS* Return to sport

### Clinical variables results

As displayed in Table 1, the clinical indicators show that the mean age is highest in the sports level-reduced group (56.18 ± 6.7) and lowest in the sports level-improved group (53.82 ± 4.4), but there is no significant statistical difference (*P* = 0.269). The BMI is highest in the sports level-reduced group (28.20 ± 6.5) and lowest in the sports level-improved group (27.31 ± 3.34), but there is no significant statistical difference (*P* = 0.614). There is a significant statistical difference in the level of preoperative sport activity among the three groups. The sports level-reduced group includes 102(64.6%) people with a low preoperative sport activity level, 12(42.9%) people with a medium preoperative sport activity level, and 10(35.7%) people with a high preoperative sport activity level. The sports level-improved group contains 32(97.0%) patients with a low preoperative sport activity level, 1(3%) patient with a medium preoperative sport activity level, and 0(0%) patient with a high preoperative sport activity level.

The results of the Mantel–Haenszel chi-square test show that there is a linear relationship between the recovery of the preoperative and postoperative activity level. The Pearson correlation result shows *χ*2 = 34.619, *P* < 0.001, indicating that the recovery of postoperative activity level decreases as the preoperative activity level increases.

There is a significant statistical difference in the distribution of preoperative Lysholm knee score in the three groups, with the average preoperative Lysholm knee score in the sports level- reduced group being the highest (60.11 ± 18.2) and the average preoperative Lysholm knee score in the sports level-improved group being the lowest (46.94 ± 14.0). Preoperative desire level for returning to sport is evenly distributed among the three groups. Although patients with extremely high make up 63.6% of the improved-sports level group, there is no significant statistical difference. In addition, there is also no significant statistical difference between patient’s gender and surgical side, with or without a meniscus surgery history, whether the breadwinner of the family and the postoperative sport level.

### Radiological variables results

We have also compared the differences among three groups of radiological indicators by ANOVA analysis, the results are presented in Table 2. As illustrated in the figure, no significant statistical differences were observed in K–L grade, HKA, MPTA, JSA, PPTA and BPI before and after surgery and preoperative %MA. However, statistical differences were found in the aspect of %MA postoperatively.

**Table 2:** Baseline of radiological variables among the sport level-reduced, unchanged and improved groups

Characteristics	Total (*n* = 158)	Sport level-reduced group (*n* = 28, 17.7%)	Sport level-unchanged group (*n* = 97, 61.4%)	Sport level-improved group (*n* = 33, 20.9%)	*P* value
K–L grade pre, *n* (%)					0.947
I	6(3.8%)	1(3.6%)	4(4.1%)	1(3.0%)	
II	123(77.8%)	21(75%)	76(78.4%)	26 (78.8%)	
III	28(17.7%)	6(21.4%)	17(17.5%)	5(15.2%)	
IV	1(0.6%)	0(0%)	0(0%)	1(3.0%)	
K–L grade post, *n* (%)					0.741
0	1(0.6%)	0(0%)	1(1.0%)	0(0%)	0
I	4(2.5%)	1(3.6%)	2(2.1%)	1(3.0%)	I
II	124(78.5%)	22(78.6%)	76(78.4%)	26(78.8%)	II
III	28(17.7%)	5(17.9%)	18(18.6%)	5(15.2%)	III
IV	1(0.6%)	0(0%)	0(0%)	1(3.0%)	IV
HKA pre	82.49 ± 4.1	81.96 ± 3.5	82.43 ± 4.4	83.09 ± 3.3	0.548
HKA Post	89.96 ± 2.9	89.71 ± 3.3	90.06 ± 2.8	89.85 ± 3.3	0.829
%MA pre	18.78 ± 15.6	14.29 ± 15.3	18.69 ± 15.9	22.88 ± 14.3	0.1
%MA post	53.35 ± 11.3	48.39 ± 12.9	54.33 ± 10.1	54.70 ± 12.3	**0.036**
LDFA pre	88.87 ± 2.0	89.36 ± 2.0	88.78 ± 2.0	88.70 ± 1.6	0.337
LDFA post	89.17 ± 1.8	89.36 ± 2.1	89.10 ± 1.8	89.21 ± 1.5	0.799
MPTA pre	83.44 ± 3.1	83.32 ± 2.7	83.30 ± 3.5	83.97 ± 2.2	0.552
MPTA Post	90.18 ± 2.9	90.04 ± 2.9	90.03 ± 3.01	90.73 ± 2.25	0.463
JLCA pre	2.85 ± 1.6	3.11 ± 1.8	2.84 ± 1.6	2.70 ± 1.4	0.592
JLCA Post	2.37 ± 1.6	2.43 ± 1.8	2.36 ± 1.6	2.36 ± 1.5	0.981
PPTA pre	81.04 ± 3.8	82.18 ± 3.6	80.62 ± 3.9	81.30 ± 3.7	0.148
PPTA post	78.85 ± 7.1	76.32 ± 14.0	79.16 ± 4.3	80.06 ± 4.18	0.094
BPI pre	1.03 ± 0.2	1.03 ± 0.3	1.01 ± 0.18	1.11 ± 1.0	0.078
BPI post	0.86 ± 0.19	0.89 ± 0.2	0.84 ± 0.19	0.90 ± 0.18	0.165

Continuous variables were described as mean ± standard and categorical variables as frequencies with percentage (%)

*HKA* hip–knee–ankle angle, %*MA* Percentage of mechanical axis, *MPTA* Percentage of mechanical axis, *JLCA* Joint line convergence angle, *PPTA* Posterior proximal tibial angle, *K–L grade* Kallgren–Lawrence osteoarthritis grade, *BPI* Blackburn–Peel index

The postoperative %MA of patients in the sports level-reduced group is 48.39 ± 12.9, the %MA post-op of patients in the sports level-unchanged group is 54.33 ± 10.1, the %MA post-op of patients in the sports level-improved group is 54.70 ± 12.3, and the difference in %MA among the three groups is statistically significant (*F* = 3.389, *P* < 0.001). According to the test, the difference in %MA post-op between the sports level-improved group and the level-reduced group is statistically significant (*P* < 0.05), while the differences in %MA post-op between the sports level-unchanged and the level-reduced group, and between the level-unchanged and the level-improved group, are not statistically significant (*P* > 0.05). Based on previous studies and clinical practice [13, 17–19], we added several factors (age, BMI, Kallgren–Lawrence osteoarthritis grade of preoperative) and carried out an ordered logistic regression. The results can be seen in Table 3.

**Table 3:** Results of ordered logistic regression analysis of sport level-reduced and improved group

	*β*	SD	*P*	OR	95%CI
%MA post	0.035	0.017	0.039	1.036	1.002–1.071
Lysholm pre	− 0.042	0.011	0.000	0.959	0.938–0.980
Age	− 0.142	0.035	0.000	0.868	0.810–0.930
BMI	− 0.131	0.048	0.006	0.877	0.798–0.964
SAL pre = 1	3.707	0.678	0.000	40.730	10.789–153.766
SAL pre = 2	0.358	0.650	0.582	1.430	0.400–5.113
SAL pre = 3	0^a^				
K–L grade pre = 1	0.007	1.037	0.995	1.007	0.132–7.683
K–L grade pre = 2	1.039	0.546	0.057	2.828	0.970–8.244
K–L grade pre = 3	0^a^				

^a^The Sport level-unchanged group was the reference group. *P < 0.05, *P < 0.01

%*MA* Percentage of mechanical axis, *Lysholm* Lysholm knee score, *SAL* Sport activity level, *K–L grade* Kallgren–Lawrence osteoarthritis grade

*CI* Confidence interval, *OR* Odds ratio

Using an ordered Logistic regression that adheres to the proportional odds assumption, the effects of age, BMI, postoperative MA%, preoperative Lysholm knee score, preoperative sport activity level and others on the postoperative sport activity level were analyzed. The result of the parallel line test is *χ*2 = 14.443, *P* = 0.209, indicating that the proportional odds assumption exists. Postoperative MA% (95%CI 1.002–1.071, *χ*2 = 0.017, *P* = 0.039) makes a significant positive impact on the postoperative sport activity level, for every unit increase, the level of postoperative movement elevates to 1.036 times its original value.

Age(95%CI 10.810–0.930, *χ*2 = 0.035, *P* < 0.001), BMI(95%CI 0.798–0.964, *χ*2 = 0.048, *P* = 0.006), preoperative Lysholm knee score(95%CI 0.938–0.980, *χ*2 = 0.011, *P* < 0.001) has a significant negative impact on the change in sport activity level before and after surgery. For each additional unit, the level of post-op movement is respectively reduced to 0.868, 0.877 or 0.959 times the original value.

Taking patients with high preoperative sport activity level as the control group, the OR value of patients with low preoperative sport activity level is 40.730 times that of patients with high preoperative sport activity level (95%CI 10.789–153.766, *χ*2 = 0.678, *P* < 0.001).

## Discussion

The major contribution of this study is the discovery that a higher postoperative MA% can facilitate the improvement of postoperative sport activity level. Meanwhile, consistent with common cognition, younger in age, lower BMI and lower preoperative Lysholm knee score are favorable factors in improving the postoperative sport activity level.

Similar to previous research outcomes, the increase in postoperative MA% can reduce the stress on the medial gap of the knee joint, alleviate preoperative knee pain, promote postoperative activity level, and slow down the progress of knee osteoarthritis. A study [4] based on the 3D finite element knee model derived from the magnetic resonance imaging of a healthy participant indicated that, a higher MA% was able to transfer the loading point to the lateral tibial plateau, with equal loading occurring at angles of 4.3° and 2.9° valgus for the femoral and tibial cartilage respectively. In addition, this research also aligns with the findings of a cadaveric study [5], that an alignment between 0° and 4° valgus was suggested to achieve a balanced stress distribution.

Similar to previous literature reports’ findings, most patients in this study returned to physical activity after HTO [20]. Patients with a greater BMI desire less physical activity, and the comparatively heavier load on the knee joint also restricts them from performing more strenuous exercises. Howells et al. [21] discovered that in cases patients with a BMI > 30, their knee scores were worse 5 years after HTO comparatively, and overweight patients may suffer a higher risk of non-union [22]. However, this conclusion is different from the findings of some other researchers [23–25], They claim that there is no correlation between BMI and postoperative outcomes after tibial tuberosity high tibial osteotomy.

In line with intuitive assessment, this study indicates that patients with a younger age, as compared to those with an older age, have a greater possibility of recovery or improvement of sport activity level. This could be ascribed to their stronger physical resilience and vibrant lifestyle. Older patients may be less keen on physical activity due to their worries over the deterioration of osteoarthritis and potentiality of knee replacement surgeries in future. They prefer reducing movement after surgery, which could be a barrier to improving sport activity level for older patients. This perspective aligns with certain prior research outcomes [17, 26]. Wang et al. has conducted a study on the factors influencing the efficacy of spacer-type medial opening wedge high tibial osteotomy(MOWHTO) in treating medial osteoarthritis of knee. The results suggest that age > 70 is a significant factor for poorer clinical outcomes after surgery. The results may be related to factors such as slower muscle strength recovery in elderly patients, longer time needed for osteotomy healing, deep vein thrombosis in the lower limbs after surgery, and decreased lung function [19]. However, the research of Kohn et al. indicates that HTO is an efficacious treatment for medial knee osteoarthritis, but the effectiveness of the treatment is not correlated with the factor of age, hence, the indications for HTO need not be concerned with issues pertaining to age [17]. Another researcher [27] have identified that the factor influencing the postoperative outcome of HTO is Cartilage Status, rather than age.

Under the same postoperative Lysholm knee score, patients with lower preoperative Lysholm knee score are better relieved from knee joint pain symptoms after surgery compared to those with higher preoperative Lysholm knee score, and have more confidence in returning to sports, thus enjoying a better chance promoting activity level. Katagiri et al.[28] also discovered that high preoperative Tegner scores were a prognostic risk factor for not returning to preoperative sports levels after open-wedge high tibial osteotomy (OWHTO).

Additionally, this study explored the impact of preoperative desire level for sport on the improvement of postoperative sport activity level, but it did not show any. This result is similar to the previous research findings of Saragaglia et al. [29]. They studied the relationship between the motivation and the resumption of physical activity and sports after medial unicompartmental osteoarthritis, and concluded that neither the motivation nor the preexistent sport level was related to better sport resumption.

This study also investigated the relationship between being the breadwinner of the family and the postoperative activity outcome and the results showed that these two events are not relevant. However, Alexander et al. [18] found that being the family's breadwinner was associated with returning to work within six months.

Besides, the results of this study suggest that preoperative and postoperative MPTA does not affect the improvement of postoperative sport activity level. A previous study suggested that a high postoperative MPTA was a prognostic risk factor for unsatisfactory outcomes in terms of returning to sports after OWHTO [28].

HTO, as a valid treatment method for KOA, is commonly deemed more applicable for younger, active patients [7, 11, 28, 30]. Some research has suggested that there’s no difference in the domain of returning to recreational activity and short-term clinical outcomes when comparing the postoperative outcomes of HTO and UKA in treating medial unicompartmental osteoarthritis [31], while other studies suggest that patients who underwent HTO were more physically active and more quicker to return to physical activity [7, 32]. In addition, Primeau et al. shows that 95% of patients who undergo HTO do not go on to have TKA within 5 years, and 79% do not go on to have TKA within 10 years [33].

Some have expressed worries about the aftermath of HTO, considering that HTO procedure has altered the structure and position of knee, hence heightening the complexity for further TKA in the future [34–36]. However, some other studies [37–40] indicates total knee arthroplasty after high tibial osteotomy approved excellent long-term survival, and could achieve the desired effect of 10-year survivorship free from loosening of 97% [41].

During HTO surgery, the gap measurement techniques used to control the alignment of lower limbs often lead to a deviation from the preoperative plan. It was reported that the surgical accuracy of using navigation equipment was about 2° deviating from the plan, which cannot be completely eliminated [42]. It may be more accurate to use an osteotomy gap measuring device. The starting-location of the osteotomy does not need to be particularly accurate to the preoperative plan, and moving distal or proximal 1 cm from initial digital plan will not affect the osteotomy gap size when correcting the alignment of lower limbs [43].

Although tibial slope did not associate with improvement on activity level after HTO in this study, it was important for knee joint. Improper fixation position and unadapted indication of bone graft could cause postoperative tibial slope be variation. [44, 45].A biomechanics analysis of fifteen sawbones tibia model indicates that, the position of the plate toward the posterior provides greater stability [16]. To maintain the stability of the osteotomy, previous literature supported that open wedge osteotomy gaps up to 14 mm without bone graft or bone substitutes could avoid unnecessary morbidity, and that the healing time is shorter when the osteotomy gap is less than 8 mm than when the osteotomy gap is wider than 14 mm [15].

Recent years, reports of treatment with cartilage restoration and repair procedures for chondral and osteochondral defects of the knee have increased, the association of arthroscopic implantation of autologous chondrocytes with a medial opening wedge osteotomy of the proximal tibia is a viable option for the management of chondral defects in varus knees [46]. Compared with isolated autologous chondrocyte implantation or osteochondral allograft transplantation, osteotomy at the time of index autologous chondrocyte implantation or osteochondral allograft transplantation procedure significantly reduces the risk of reoperation without increasing the risk, complications and medical cost [47].

### Limitation

Several limitations of our study may have influenced the results. First, the unequal number of compared groups limited the research on patients’ sport activity level after HTO, because the general population included in this study has a lower proportion of participation in high-level sports before surgery. Second, influenced by public perception, many older patients’ postoperative Lysholm knee score has significantly improved compared to pre-surgery ones, but they do not wish to participate in higher-level sports due to their physical endurance concerns, which may not fully reflect the real improvement in their postoperative sport activity level. At last, this study is a single-center research, and the limited number of participants as well as the local population's concept of physical activity may have an impact on the research outcomes.

## Conclusion

This study found that an increase in MA% after surgery, younger age, lower BMI, and lower postoperative Lysholm knee score contribute to the improvement of postoperative sport activity level. The study provides guidance for postoperative MA% goals in clinical practice and offers predictions for postoperative sport activity level evaluation of different populations undergoing HTO surgery, also makes references in setting rehabilitation plan.

## Data Availability

All data and materials are available from the corresponding author upon request.

## Abbreviations

OA: Osteoarthritis
KOA: Knee osteoarthritis
HTO: High tibial osteotomy
MOWHTO: Opening wedge high tibial osteotomy
OWHTO: Open-wedge high tibial osteotomy
HKA: Hip–knee–ankle angle
%MA: Percentage of mechanical axis
MPTA: Percentage of mechanical axis
JLCA: Joint line convergence angle
PPTA: Posterior proximal tibial angle
K–L grade: Kallgren–Lawrence osteoarthritis grade
BPI: Blackburn–Peel index
BMI: Body mass index
SAL: Sport activity level
CMT: Concomitant meniscal treatment
Lysholm: Lysholm knee score
RTS: Return to sport
